# How does rural population aging affect agricultural green development in China?

**DOI:** 10.1371/journal.pone.0333370

**Published:** 2026-05-26

**Authors:** Lina Xu, Zhaozhe Teng, Yin Liu, Weizhong Liu

**Affiliations:** 1 School of Economics and Management, Nanjing Agricultural University, Nanjing, Jiangsu, China; 2 School of Economics and Management, Xinjiang Agricultural University, Urumqi, Xinjiang, China; Università degli Studi di Milano: Universita degli Studi di Milano, ITALY

## Abstract

Rapid population aging significantly impacts socioeconomic development and poses substantial challenges for agricultural green development (AGD), which have so far not been well understood. This study uses data from the China Family Panel Studies conducted in 2018, 2020, and 2022 and adopts the instrumental variable two-stage least squares (IV-2SLS) method to analyze the impact and mechanisms of rural population aging on AGD from a micro-level perspective of farmers. The benchmark regression results show that rural population aging significantly hinders AGD: an increase in the proportion of older adults in the household reduces AGD by 0.346. The impact in the western region is greater than that in the central and eastern regions, primarily by restricting improvements in economic benefits (agricultural output). In contrast, its effect on environmental benefits (agricultural chemical inputs) is not statistically significant. The adoption of agricultural socialized services by older adults increases agricultural output while reducing chemical inputs, thereby mitigating the adverse effects of rural population aging on AGD. However, surging service prices discourage utilization, prompting farmers to transfer or abandon part of their farmland and continue traditional resource-intensive practices. Our analysis indicates that it is crucial to establish mechanisms to curb farmland abandonment and enhance the accessibility of agricultural socialized services for older adults in the context of rapid population aging.

## 1. Introduction

Agricultural environmental pollution and a shortage of production resources have become urgent issues hindering global development [1–3]. Ensuring safe agricultural production that does not damage the environment has become an important concern for governments worldwide [4,5]. China feeds over 1.4 billion people (nearly 18% of the global population) with only 9% of the world’s arable land [6]. Driven by the policy goal of “increasing production and income,” Chinese agriculture has long relied on resource-intensive farming practices. Data from the “China Ecological Environment Status Bulletin 2020” show that the fertilizer and pesticide utilization rates for China’s major staple crops (rice, wheat, and maize) were only 40.2% and 40.6%, respectively, in 2020. This strategy of increased production wastes resources and leads to nutrient loss, further deteriorating the environment and directly harming human health [7,8]. To address this, the Chinese government has vigorously promoted agricultural green development (AGD) since 2017, listing it as a key task in advancing the Rural Revitalization Strategy [9]. In essence, AGD is a form of high-quality sustainable development. It is a development mode that aims to achieve optimal outputs and comprehensive benefits with minimal inputs and environmental costs.

The aging of the agricultural labor force has become a prominent issue in China [10–12]. Data from the China Household Finance Survey indicate a steady increase in the share of agricultural workers aged 60 and older, rising from 37.35% in 2015 to 39.46% in 2017, 42.13% in 2019, and 43.04% in 2021. Given the persistently declining birth rate and shrinking agricultural labor pool, the aging trend of China’s agricultural workforce is expected to intensify further in the future.

Rural population aging can lead to severe labor shortages and human capital constraints, potentially impacting AGD. Zagata and Sutherland [13] examined European agriculture and found that agricultural performance was more severely affected by aging in countries with a higher proportion of smallholders. Given the rapid aging of the rural population and the prevalence of smallholders in China, concerns have risen that an aging rural population may lead to a decline in agricultural productivity, thereby threatening food security and AGD.

Current academic discussions on the impact of aging on agricultural production predominantly focus on agricultural output [14–19] or green technology adoption [20–23] while overlooking holistic sustainable production capacity. Undoubtedly, a complex trade-off exists between desirable output and environmental pollution. A critical challenge lies in finding a balance between maximizing agricultural output and mitigating environmental pollution. Green agricultural development requires a balanced approach reconciling productivity enhancement and ecological conservation. It emphasizes improving agricultural green total factor productivity (AGTFP) to ensure the stability and sustainability of the food supply [24,25]. Improving AGTFP is a key measure for AGD in developing countries [26,27].

Six recent studies closely related to this research are worthy of discussion. First, Li et al. [28] demonstrated the dual temporal effects of population aging on AGTFP, showing initial suppression followed by long-term improvement through knowledge spillovers and industrial restructuring. Second, Jin and Wang [29] identified a significant positive correlation between aging and AGTFP, mainly because older adults tend to transfer their farmland to more productive new agricultural entities. In contrast, Du et al. [30], Song et al. [31], and Liu et al. [18] reached opposite conclusions, finding a significant negative correlation. They argued that older adults restrict the application of new technologies in agricultural production. These studies have primarily focused on macro-level analysis, using aging ratios (the proportion of rural populations over 60 years old at provincial-level), but ignore the fact of labor mobility between urban and rural areas, resulting in biased indicator measurement. Moreover, since agricultural production in China is still predominantly family-based, households with varying ratios of aging laborers often exhibit distinct behavioral preferences and objectives. This has led to diverse factor allocation and adoption strategies. Hence, conducting a differentiated assessment of agricultural green production practices by aging households is necessary to accurately evaluate the impact of rural population aging on AGD. Although Ren et al. [14] used micro-level data from a survey of over 30,000 households and found that rural population aging led to a 4% decline in agricultural output and an increase in fertilizer-related pollutant emissions, their study did not extend to an analysis of AGTFP.

The aforementioned research offers valuable insights for this study. However, existing research has not yet fully elucidated the impact of rural population aging on AGTFP. The reasons for this are as follows: First, the existing research has primarily focused on the provincial macro-level, lacking in-depth exploration of individual older adults behaviors, particularly the differences in agricultural chemical inputs. This makes it more difficult to accurately grasp the real situation in rural areas. Second, most existing research overlooks the impact of households that have exited (even temporarily) agriculture but still hold agricultural production factors. Therefore, it is essential to consider the issue of self-selection in labor allocation. Third, existing research generally assumes that older adults alleviate physical and human capital constraints in the labor force by transferring farmland to new agricultural business entities. However, older adults account for 41.04% of all agricultural laborers and remain an important force in agricultural production in China. Therefore, exploring the agricultural production characteristics of older adults is of practical importance. Fourth, developing socialized services is an effective way to mitigate the negative impact of aging on agricultural output. However, in the context of rising service prices and low-profit agricultural operations, the adoption behaviors of older adults and their impact on AGD need to be reexamined [32].

The marginal contributions of this study are threefold: (1) Pioneering a micro-level investigation of the impact and mechanisms of rural population aging on AGD, thereby complementing conventional macro-level analyses. Unlike prior macro-level studies, this study focuses on individual farmers’ production practices. This fills the gap in existing literature regarding the micro-level mechanisms. (2) Assessing the impact of rural population aging on AGD through dual analytical dimensions: economic benefits (agricultural output) and environmental benefits (agricultural chemical inputs) in agricultural production, this study enriches research practices that coordinate economic-environmental synergies in agricultural growth from an aging perspective. (3) By integrating the mediating role of agricultural socialized services into the analytical framework and considering the impact of rural population aging on farmland allocation, this study comprehensively explores the mechanism of the impact of aging on AGD, and broadens the scope of research in this field.

This paper is organized as follows. Section 2 presents the theoretical analysis and research hypotheses. Section 3 describes the measurement of AGD and the research design. Empirical results are provided in Section 4. Section 5 discusses the findings. Finally, Section 6 presents conclusions and policy implications.

## 2. Theoretical analysis and research hypothesis

### 2.1. Improvement *in* AGTFP and agricultural green development

Currently, there is no consensus in academia regarding the definition of AGD. We contend that improving AGTFP is highly consistent with the objectives and strategies of AGD as outlined in the “Guidelines for Agricultural Green Development (2018-2030)” issued by the Ministry of Agriculture and Rural Affairs in China. Achieving AGD fundamentally hinges on steadily improving AGTFP [33]. This alignment can be explained through three aspects: (1) AGD requires a shift in focus from quantity to an emphasis on balancing quantity and quality. AGTFP calls for a change in China’s long-standing extensive agricultural production model, optimizing input allocation efficiency, and pursuing an element-intensive approach that enhances quality and efficiency. The goal is to achieve optimal agricultural output, efficiency, and returns with minimal resource input and environmental costs [34]. Thus, improvements in AGTFP and AGD are inherently unified. (2) Green agricultural development calls for a shift in focus from production functions to an equal emphasis on ecological functions. AGTFP integrates economic benefits with environmental costs, incorporating green production technologies and practices such as formula fertilization after soil testing and green pest control. These practices reduce the ecological damage caused by agricultural production under the AGD concept of “economic growth with reduced pollution emissions.” (3) AGD requires a shift in focus from improving single-factor productivity to improving total factor productivity. This shift aligns closely with the essence of AGTFP improvement. As a quantifiable indicator, AGTFP measures the efficiency of agricultural input utilization and reveals the comprehensive efficiency of AGD. This reflects the current level of agricultural modernization and aligns with the overall goals of AGD. In summary, AGTFP is an ideal measure of AGD.

### 2.2. Theoretical analysis of the impact of rural population aging on AGD

Within the AGTFP conceptual framework, AGD aims to minimize environmental pollution while ensuring the stability or continuous growth of agricultural outputs. Therefore, the impact of rural population aging on AGD can be analyzed by examining its effects on both economic (agricultural output) and environmental benefits (agricultural chemical inputs).

The impact of rural population aging on economic benefits is called the output effect. Grounded in Lucas (1988), the endogenous growth theory posits that the stock of human capital is the engine of long-term economic growth [35]. Building on Schultz and Becker’s ideas, Lucas assumed that the growth of human capital stock depends on both existing stock and new investments in human capital, concluding that human capital growth leads to technological innovation and improvements in labor productivity. However, in the context of population aging, insufficient new human capital severely affects the accumulation of human capital stock. In this scenario, farmers must reallocate labor between agricultural and non-agricultural sectors to maximize the returns from labor allocation. Economic agents aiming to optimize labor resource allocation tend to assign younger laborers to non-agricultural sectors, where labor productivity is typically higher. This has led to a reduction in the number of agricultural laborers. Moreover, older adults, who are at a clear disadvantage in terms of health and labor intensity, exhibit reduced labor quality. The decline in the quality of the agricultural labor force directly affects both the amount of labor input and the level of intensive cultivation in agricultural production, hindering an increase in economic benefits.

The impact of rural population aging on environmental benefits is known as the environmental effect. Agricultural non-point source pollution is a key challenge in eight major pollution control battles in China. Induced technological change theory elucidates farmers’ rational economic responses to farmland constraints, as they rely on agrochemical intensification to maximize economic benefits. However, the ecological consequences of this practice have created a dual dilemma: ecosystem degradation and compromised food safety. To address this issue, the Chinese government has focused on prominent environmental issues related to agricultural production. For example, to reduce chemical fertilizer usage, local authorities have continuously introduced efficiency-enhancing alternatives such as soil testing and formula-based fertilization, organic fertilizer substitution, and integrated water and fertilizer management. However, older adults show little enthusiasm for new technologies [20]. The reason is that, compared to younger farmers, older adults, while more experienced, often have outdated knowledge systems and weaker cognitive and learning abilities. Generally, adopting a new technology involves several stages, including awareness, evaluation, decision-making, adoption, and feedback. However, older adults face significant disadvantages in their cognitive abilities [36,37] and learning capacity [38] compared to younger ones. These limitations reduce their ability to learn and apply green production technologies effectively. Based on the above analysis, we propose the following hypotheses:

**H1:** Rural population aging has a negative impact on agricultural green development.

**H2:** Rural population aging impedes the improvement of economic and environmental benefits, thereby hindering agricultural green development.

Older adults often compensate for insufficient labor input by adopting agricultural socialized services. Agricultural socialized services refer to a specialized service system provided by multiple subjects, covering the entire process of agricultural production. Its core goal is to connect smallholder farmers with modern agriculture, reduce production and operation costs through resource integration and economies of scale, and promote the intensive and green development of agriculture [39–41]. We focused primarily on two types of services: machinery rental and labor hiring. older adults frequently hire labor and utilize agricultural mechanization services such as fertilization and pesticide spraying, generating a steady demand for services [42]. This can reshape their production behavior [43].

The adoption of agricultural socialized services by older adults improves economic benefits. Mechanization has been widely adopted in the key labor-intensive stages of crop production, significantly reducing the physical demands on laborers. According to a survey by the U.S. Department of Agriculture, the average age of the 2 million agricultural workers in the U.S. is 55, yet agricultural mechanization enables them to handle the physical demands of various farm tasks. This indicates a strong substitutive effect of machinery on labor, in which mechanization significantly reduces laborers’ physical constraints, thereby mitigating the inhibitory effects of aging on agricultural output.

The adoption of agricultural socialized services by older adults improves economic benefits by reducing the use of chemical fertilizers and pesticides [44]. First, compared to individual farmers, socialized service providers are better able to collect and discern information on fertilizer and pesticide efficacy, reducing overapplication. Second, the Chinese government is increasingly prioritizing the provision of socialized services for agricultural green production, such as deep plowing, straw incorporation, formula fertilization after soil testing, and green pest control [45]. Service providers incorporate green technologies into their offerings to obtain policy support, such as project and service subsidies [46]. In addition, older adults can improve the efficiency of agricultural chemical applications by utilizing mechanization services. For instance, substituting manual fertilization with mechanical fertilization can mitigate the uneven and irregular application associated with manual methods, thereby improving fertilizer efficiency and avoiding unnecessary overapplication. Based on the above analysis, we propose the following hypothesis:

**H3:** The adoption of agricultural socialized services by older adults improves economic and environmental benefits, thereby mitigating the adverse effects of rural population aging on agricultural green development.

## 3. Materials and methods

### 3.1. Data sources

The data used in this study were obtained from the CFPS database created by Peking University. The CFPS data have been anonymized and comply with relevant ethical guidelines for the use of public research data. The survey covers households and all family members in 25 provinces, municipalities, and autonomous regions in China, providing a reliable representation of the country’s social, economic, and demographic characteristics. Since the most recent village-level survey in the CFPS was conducted in 2014, and later waves (2018–2022) contain only individual- and household-level data, we matched the 2018–2022 household data with the 2014 village data to incorporate key village-level variables: distance to the county seat and presence of high-polluting enterprises. This matching assumes these characteristics remained relatively stable over the study period, for two key reasons. First, distance to the county seat is a fixed geographical feature tied to infrastructure, which rarely undergoes fundamental changes in the short term in rural China, particularly outside metropolitan fringe areas. Second, the presence of high-polluting enterprises in villages is subject to stringent environmental regulations and industrial planning; therefore, the entry and exit of such enterprises in rural areas are highly regulated, with little random fluctuation over a short period. For sample selection, we examined the impact of rural population aging on AGD. Therefore, the study retained households engaged in farming, forestry, animal husbandry, sideline, or fishery activities, with at least one farming laborer aged 60 or above. The final analytical sample constituted a balanced short panel covering three survey waves, with a total of 1479 household-year observations.

### 3.2. Measurement of crucial variables

#### 3.2.1. Dependent variables.

AGTFP involves multiple inputs and outputs, including desirable and undesirable outputs, making it unsuitable for measurement using parametric methods based on production function construction. Therefore, we utilized the Slack Based Measure-Global Malmquist-Luenberger (SBM-GML) index, which incorporates undesirable outputs, to construct an AGTFP indicator for measuring agricultural development at the household level. This method overcomes the limitation of efficiency values being capped at one and addresses the issue of variable slack, resulting in more scientifically robust and reliable measurement outcomes. Therefore, with reference to the study of Tone [47], we calculate AGTFP using the following formula:


$$\begin{array}{c} \rho =\text{min}\frac{\text{1}-\frac{\text{1}}{\text{m}}\sum_{\text{i}=\text{1}}^{\text{m }}\frac{\overset{-}{\underset{\text{i}}{\text{s}}}}{{\text{x}}_{\text{i}}}}{\text{1}+\frac{\text{1}}{{\text{s}}_{\text{1}}+{\text{s}}_{\text{2}}}(\sum_{\text{k}=\text{1}}^{{\text{s}}_{\text{1}}}\frac{\overset{\text{g}}{\underset{\text{r}}{\text{s}}}}{\overset{\text{g}}{\underset{\text{r}}{\text{y}}}}+\sum_{\text{k}=\text{1}}^{{\text{s}}_{\text{2}}}\frac{\overset{\text{b}}{\underset{\text{k}}{\text{s}}}}{\overset{\text{b}}{\underset{\text{k}}{\text{y}}}})} \end{array}$$ (1)

$$\begin{array}{c} \text{s}.\text{t}.{\text{x}}_{\text{0}}=\lambda \text{x}+{\text{s}}^{-} \end{array}$$

$$\begin{array}{c} \text{s}.\text{t}.\overset{\text{g}}{\underset{\text{0}}{\text{y}}}=\lambda {\text{y}}^{\text{g}}-{\text{s}}^{\text{g}} \end{array}$$

$$\begin{array}{c} \text{s}.\text{t}.\overset{\text{b}}{\underset{\text{0}}{\text{y}}}=\lambda {\text{y}}^{\text{b}}-{\text{s}}^{\text{b}} \end{array}$$

$$\begin{array}{c} {\text{s}}^{-}\geq \text{0},{\text{s}}^{\text{g}}\geq \text{0},{\text{s}}^{\text{b}}\geq \text{0} \end{array}$$

In Equation (1), ρ denotes the efficiency of a decision-making unit (DMU). A value of ρ≥1 indicates an efficient DMU, while ρ<1 implies the presence of inefficiency, warranting further improvements to the DMU’s input and output allocation.m, ${\text{s}}_{\text{1}}$ and ${\text{s}}_{\text{2}}$ represent the number of input indicators, desirable output indicators, and undesirable output indicators, respectively. k refers to a specific DMU. $\overset{-}{\underset{\text{i}}{\text{s}}}$ and ${\text{x}}_{\text{i}}$ denote input slack and input variables, respectively. x, ${\text{y }}^{\text{g}}$ and ${\text{y }}^{\text{b}}$ are the values of input, desirable output, and undesirable output variables required for DMU evaluation. ${\text{s}}^{-}$, ${\text{s }}^{\text{g}}$ and ${\text{s }}^{\text{b}}$ represent input slack, desirable output shortfall, and undesirable output surplus, respectively. $\overset{\text{g}}{\underset{\text{r}}{\text{s}}}$ and $\overset{\text{g}}{\underset{\text{r}}{\text{y}}}$ denote the shortfall and variable of the r-th desirable output, respectively; $\overset{\text{b}}{\underset{\text{k}}{\text{s}}}$ and $\overset{\text{b}}{\underset{\text{k}}{\text{y}}}$ denote the surplus and variable of the k-th undesirable output, respectively. λ is the weight vector.

To enable intertemporal comparison of productivity, we adopt the GML index to measure the dynamic changes in AGTFP over the research period with reference to the study by Ma et al. [48], and the calculation formula is as follows:


$$\begin{array}{c} {\text{GML}}^{\text{t},\text{t}+\text{1}}({\text{x}}^{\text{t}},{\text{y}}^{\text{t}},{\text{b}}^{\text{t}},{\text{x}}^{\text{t}+\text{1}},{\text{y}}^{\text{t}+\text{1}},{\text{b}}^{\text{t}+\text{1}})=\frac{\text{1}+\overset{\text{T}}{\underset{\text{G}}{\text{D}}}({\text{x}}^{\text{t}},{\text{y}}^{\text{t}},{\text{b}}^{\text{t}})}{\text{1}+\overset{\text{t}+\text{1}}{\underset{\text{G}}{\text{D}}}({\text{x}}^{\text{t}+\text{1}},{\text{y}}^{\text{t}+\text{1}},{\text{b}}^{\text{t}+\text{1}})}\; \end{array}$$ (2)

In Equation (2), x denotes input factors; y denotes desirable outputs; b denotes undesirable outputs. $\overset{\text{T}}{\underset{\text{G}}{\text{D}}}({\text{x}}^{\text{t}},{\text{y}}^{\text{t}},{\text{b}}^{\text{t}})$ and $\overset{\text{t}+\text{1}}{\underset{\text{G}}{\text{D}}}({\text{x}}^{\text{t}+\text{1}},{\text{y}}^{\text{t}+\text{1}},{\text{b}}^{\text{t}+\text{1}})$ represent the global directional distance functions for period t and period t + 1, respectively. ${\text{GML}}^{\text{t},\text{t}+\text{1}}$ is the efficiency value of period t + 1 relative to period t; a value of ${\text{GML}}^{\text{t},\text{t}+\text{1}}>\text{1},<\text{1},$ or =1 indicates an increase, decrease, or no change in efficiency from period t to period t + 1, respectively.

We constructed a scientific and multi-dimensional AGTFP measurement index system (Table 1) to quantify agricultural green development at the household level [49,50]. A core challenge to measuring AGTFP at the micro level is the lack of farm household-level data on undesirable outputs. To address this, we adopted a dual-measurement framework for quantifying these outputs in AGTFP calculations: we used objective provincial-level environmental pollution indicators as the primary measure, and household heads’ subjective environmental perceptions as the supplementary measure. We further conducted empirical analysis and robustness tests based on these two measures to mitigate potential measurement bias. As shown in Table 1, the primary undesirable output measure uses objective provincial-level agricultural non-point source pollution data, including chemical oxygen demand, total nitrogen, and total phosphorus emissions from agricultural production. This objective measure serves as the core basis for our main empirical analysis because it directly reflects the actual environmental impact of agricultural production activities and avoids the interference of non-agricultural factors such as nearby industrial pollution on the measurement results. The supplementary undesirable output measure is the household head’s perceived severity of environmental pollution, scored from 0 (not serious) to 10 (very serious), which we used as an alternative indicator for robustness verification. The household head’s perception may be influenced by non-farm factors such as regional industrial pollution and natural environmental changes, which could introduce minor subjectivity bias and measurement error. To mitigate such issues, we controlled for village-level variables such as the presence of high-polluting enterprises in all regression models to exclude the interference of non-agricultural pollution sources on the subjective perception measure.

**Table 1:** The input-output indicator system of agricultural green total factor productivity (AGTFP).

Items	Factor indicators	Definition	Unit
Input factors	Capital input	Total investment in fluid capital and fixed capital	10^4^ yuan (RMB)
	Labor input	Total agricultural labor force	person
	Land input	Total contracted and rented land area	mu
Output factors	Desirable output	Primary industry output	10^4^ yuan (RMB)
	Undesirable output1	Chemical oxygen demand	10^4^ t
			Total nitrogen
			Total phosphorus
	Undesirable output2	Perceived severity of environmental pollution by the household head (0 = not serious, 10 = very serious)	score

#### 3.2.2. Independent variables.

Rural population aging reflects the age distribution of household members engaged in agricultural production. Yang et al. [51] measured the aging index using a dummy variable for older adults and assigned values based on whether the proportion of older adults exceeded the average. However, this approach may lead to the loss of important information regarding the aging of the agricultural population. Therefore, we used the proportion of elderly farmers relative to the total number of farmers in a household to measure the aging level of the rural population. In this context, elderly farmers are defined as those aged 60 or above, with no distinction between male and female aging standards.

#### 3.2.3. Mechanism variables.

To examine the transmission mechanisms through which rural population aging affected agricultural green development (AGD), this study introduced a set of mechanism variables capturing different pathways of influence. In the empirical framework, rural population aging was the core independent variable, and AGTFP was the baseline dependent variable. Control variables were included in the benchmark model to account for other observable factors that might affect AGD, whereas the mechanism variables introduced in this subsection were used to identify how rural population aging affected AGD through different transmission channels. Specifically, economic benefits and environmental benefits were used to examine the direct channels proposed in Hypothesis 2, while agricultural socialized services were used to capture the behavioral mechanism proposed in Hypothesis 3.

**Economic benefits.** Economic benefits were quantified as income from the sale of agricultural products, livestock, and by-products, as well as the total value of self-consumed goods.

**Environmental benefits.** Because environmental pollution is primarily caused by chemical inputs and in light of the availability of micro-level data, we measured the environmental effect by calculating the proportion of seed, pesticide, and fertilizer expenses in total agricultural capital inputs. It is important to note that farmers in the same village generally use the same types of fertilizers and pesticides, and there is little price variation. Therefore, this measure is both reasonable and feasible [52].

**Agricultural socialized services.** We focused primarily on two types of services in the mid-production phase (i.e., production activities between sowing and harvesting): machinery rental and labor hiring. These services are more important and concentrated in the production process compared to pre- and post-production services, and they represent areas with the highest demand from farmers. As these absolute indicators are not directly comparable, relative indicators were used to reflect differences in the adoption of agricultural socialized services among farmers. Consequently, the proportion of labor hiring and machinery rental expenses relative to total agricultural capital inputs was used as a measure.

#### 3.2.4. Control variables.

Drawing on the existing literature related to family agricultural production and rural population aging, this study incorporates three sets of control variables, which are categorized at the individual, household, and village levels. Individual-level controls include gender, age, education level, marital status, party members, and receive pensions. Household-level controls cover labor input, the proportion of agricultural income and per capita income. Village-level controls include the distance of the village from the nearest town and the presence of polluting enterprises.

Table 2 presents the descriptive statistics of the key variables. AGTFP1 (mean = 1.281), calculated with objective provincial-level agricultural non-point source pollution as the undesirable output, is the primary indicator for our baseline empirical analysis; AGTFP2 (mean = 1.183), using the household head’s subjective environmental perception as the undesirable output, serves as the alternative indicator for robustness tests of the main conclusions. This suggests that AGD is experiencing positive growth, which supports the validity of the indicator construction and method selection. The mean value of rural population aging is 75.174%, which is a result of our selective sampling of farm households with at least one farming laborer aged 60 or above. This aligns with China’s agricultural labor aging reality: with the massive transfer of young and middle-aged rural labor to non-agricultural sectors, the proportion of elderly laborers in agricultural production of farm households maintaining agricultural operations is significantly higher. Thus, this value is a realistic reflection of the main agricultural production subjects in rural China under the background of population aging. The mean total value of household agricultural and sideline products was 13030 yuan, implying that aging farming households tend to have smaller agricultural production scales, which aligns with the realities of rural China.

**Table 2:** Descriptive statistics for the variables.

Variable	Definition	Obs.	Mean	Std.	Skewness	Kurtosis
**Dependent variable**						
Agricultural green development	AGTFP1, agricultural non-point source pollution as an undesirable output	986	1.281	0.857	0.590	2.581
	AGTFP2, household heads’ subjective perception of pollution as an undesirable output	986	1.183	0.866	0.342	2.389
**Independent variable**						
Rural population aging	Proportion of elderly farmers relative to the total number of farmers in the household	1479	75.174	27.768	−0.402	1.509
**Mechanism Variable**						
Economic benefits	Primary industry output/10^4^ yuan (RMB)	1479	1.303	1.823	0.400	1.902
Environmental benefits	Proportion of seed, pesticides, and chemical fertilizer expenses in total agricultural capital investment	1479	0.336	0.390	0.715	2.374
Agricultural socialized services	Proportion of labor hiring and machinery leasing in total agricultural capital investment	1479	0.123	0.142	0.920	2.905
**Control variable**						
Gender	Male = 1, Female = 0	1479	0.715	0.451	−0.954	1.910
Age	Years	1479	62.072	11.228	−1.488	4.751
Education	Years of schooling/Year	1479	5.361	4.415	0.074	1.871
Marital status	In marriage = 1, Other = 0	1479	0.888	0.315	−2.467	7.089
Party members	Yes = 1, No = 0	1479	0.103	0.304	2.616	7.844
Receive pensions	Yes = 1, No = 0	1479	0.732	0.443	−1.049	2.100
Labor input	Total agricultural labor force/person	1479	2.245	1.128	1.808	8.945
Share of agricultural income	Agricultural income as a proportion of total income	1479	0.442	0.349	0.435	1.706
Per capita income	10^4^ yuan (RMB)	1479	0.923	1.382	0.647	2.763
Town distance	hour	1479	4.036	7.113	−0.953	2.628
Polluting enterprises exist in the village	Yes = 1, No = 0	1479	0.150	0.357	1.959	4.838

### 3.3. Model setup

Based on the above theoretical analysis, we constructed the following econometric model to test Hypothesis 1 by examining the impact of rural population aging on AGD:


$$\begin{array}{c} {\text{agtfp}}_{\text{it}}=\alpha_{\text{1}}+\alpha_{\text{2}}{\text{aging}}_{\text{it}}+\alpha \sum {\text{controls}}_{\text{it}}+\mu_{\text{i}}+\delta_{\text{t}}+\epsilon_{\text{it}} \end{array}$$ (3)

where i represents individuals and t represents time, ${\text{agtfp}}_{\text{it}}$ represents AGD, aging represents rural population aging, and ${\text{controls}}_{\text{it}}$ is a vector of control variables. $\mu_{\text{i}}$ and $\delta_{\text{t}}$ represent individual and time fixed effects, respectively. Finally, $\epsilon_{\text{it}}$ is the random error term. To test Hypothesis 2, we estimated mechanism models in which AGTFP was replaced by economic benefits and environmental benefits, respectively, in order to examine the direct channels through which rural population aging affected agricultural green development. To test Hypothesis 3, we further examined whether rural population aging affected the adoption of agricultural socialized services, thereby capturing the behavioral mechanism described in Hypothesis 3.

The estimation of Equation (3) may suffer from endogeneity issues for the following reasons. First, the proportion of elderly farmers reflects households’ labor allocation decisions, and AGTFP also depends on labor allocation, as it integrates labor, capital, and technological efficiency. Second, reverse causality may exist because AGTFP fluctuations may induce behavioral adjustments in labor allocation strategies, creating a self-selection bias. We used an instrumental variable approach to estimate the impact of rural population aging on AGD to address these issues. We drew on existing research and selected the following two instrumental variables: 1) The proportion of older adults in the household [32,53]. Household age structure is determined by historical fertility decisions, which makes this variable predetermined and exogenous to current agricultural green production choices. Although the household elderly share may influence agricultural green total factor productivity (AGTFP) through non-labor channels (e.g., health status, caregiving burdens, or household savings), we address this concern by including individual-level controls (e.g., age, pension receipt) and household-level controls (e.g., income, labor input). These covariates absorb the direct effects of confounding factors and strengthen the validity of the exclusion restriction. 2) The village-level proportion of older adults in 2012, which serves as an instrument for household-level rural population aging in 2022. This instrument satisfies the relevance assumption because rural population aging exhibits strong temporal persistence and path dependence: the 2012 village-level age structure is strongly correlated with the 2022 household labor composition, reflecting the lasting influence of historical demographic patterns. Regarding exogeneity, the 2012 village-level elderly proportion is a historical outcome determined by pre-2012 demographic trends, rural–urban migration, and fertility decisions, and therefore cannot be affected by households’ 2022 agricultural green production decisions (e.g., AGTFP, chemical input intensity, or adoption of agricultural services). A contemporaneous village-level aging measure could influence household AGTFP through general equilibrium channels such as local labor market conditions, agricultural service availability, or land rental markets. However, the decade-long lag between the instrument (2012) and the outcome variable (2022) mitigates this concern: such general equilibrium effects would need to operate with a substantial lag and are unlikely to be directly driven by a historical demographic measure.

## 4. Results

### 4.1. Baseline regression results

We used a panel IV-two-stage least squares (2SLS) fixed-effects model to regress Equation (3) [54]. The regression results are shown in Table 3. As shown in Column (1) of Table 3, when using the proportion of older adults in the household as an instrumental variable in the regression analysis, the Kleibergen-Paap rk LM statistic was 120.67, indicating no under-identification issue. The Kleibergen-Paap rk Wald F statistic was 232.51, suggesting that the model did not suffer from weak instruments, thereby confirming the validity of the selected instrumental variable. Similarly, the corresponding Kleibergen–Paap rk LM and Wald F statistics indicate that village-level rural population aging also constitutes a valid instrumental variable. In addition, we employed a 10-year lagged indicator of rural population aging as an instrumental variable, specifically using the village-level rural population aging rate in 2012 as an instrument for the household-level rural population aging in 2022. The empirical analysis was conducted using a cross-sectional two-stage least squares (2SLS) regression model, and the results are presented in Column (2) of Table 3. As shown in Table 3, when estimations are performed using these two instrumental variables, rural population aging has a significantly negative impact on AGTFP at least at the 10% significance level. This finding indicated that the level of AGD decreases as the proportion of older adults in households increases, confirming Hypothesis 1. Several studies have proposed that aging has forced technological innovation in agriculture [43]. However, population aging, which is characterized by a reduction in the supply of young and middle-aged labor, leads to insufficient agricultural labor input. This hampers the adoption and promotion of advanced farming methods and new green production technologies, thereby directly hindering AGD.

**Table 3:** Benchmark regression results.

Variable	(1)	(2)
IV: Proportion of older adults in the household	−0.346 ***	
	(0.111)	
IV: Village-level aging rate in 2012		−0.022*
		(0.011)
Control Variables	YES	YES
Individual fixed effects	YES	
Time fixed effects	YES	
KP-LM statistic	120.670***	
KP-F statistic	232.510	
Observations	986	364

**Notes:** ***, **, and *, respectively, indicate that the coefficients are significant at the levels of 1%, 5%, and 10%. Robust standard errors are in parentheses.

### 4.2. Robustness check

We used the following two robustness testing methods on the empirical results. First, we replaced the dependent variable metric by recalculating AGTFP with the household head’s perceived severity of environmental pollution as an undesirable output. Second, the age range of the study subjects was narrowed by defining aging as individuals aged 65 or above. This was because people aged ≥65 often face more significant health challenges and greater constraints in labor supply capacity and cognitive flexibility to adopt new green production technologies, hindering agricultural green development. We used the proportion of older adults in the household as the instrumental variable to address potential endogeneity issues and conducted a 2SLS estimation across both robustness checks, as presented in Table 4. As shown, rural population aging exerted statistically significant dampening effects on AGTFP, confirming the robustness of the study’s conclusions.

**Table 4:** Regression results of robustness test.

Variable	Substitute for the Explained Variable		Set the Aging Threshold at 65 Years	
	(1)	(2)	(3)	(4)
IV: Proportion of older adults in the household	−0.233		−0.006***	
	(0.131)		(0.002)	
IV: Village-level aging rate in 2012		−0.023*		−0.022*
		(0.012)		(0.013)
Control Variables	YES	YES	YES	YES
Individual fixed effects	YES		YES	
Time fixed effects	YES		YES	
KP-LM statistic	122.869***		122.000***	
KP-F statistic	236.368		723.976	
Observations	986	364	798	364

Notes: ***, **, and *, respectively, indicate that the coefficients are significant at the levels of 1%, 5%, and 10%. Robust standard errors are in parentheses.

### 4.3. Estimation results for impact pathways

#### 4.3.1. Economic effect or environmental effect.

Based on the above theoretical analysis, the impact of rural population aging on AGD has economic and environmental benefits. A key question is what drives the inhibitory effect—changes in economic benefits, environmental benefits, or both? To address this, we re-estimated the mechanism effects using our IV–2SLS framework, with the results reported in Table 5. In Column (1) of Table 5, where the dependent variable is agricultural output (a core measure of economic benefits), the coefficient of rural population aging is −0.006 and significant at the 1% level. This indicates that a one-percentage-point increase in rural population aging is associated with a 0.6% reduction in agricultural output, which directly reflects the negative impact of aging on the economic dimension of AGD. In Column (2), where the dependent variable is agricultural chemical inputs (a proxy for environmental benefits), the coefficient of rural population aging is 0.004 and statistically insignificant. This suggests that aging does not have a measurable effect on the environmental dimension of AGD in our sample. This indicates that the inhibitory effect of rural population aging on AGD mainly originates from its negative impact on economic benefits.

**Table 5:** Regression results of mechanism analysis: Output effect or environmental effect.

Variable	Agricultural Output	Chemical Inputs
	(1)	(2)
Rural population aging	−0.006***	0.004
	(0.002)	(0.048)
Control Variables	YES	YES
Individual fixed effects	YES	YES
Time fixed effects	YES	YES
Constant	0.651***	0.059
	(0.202)	(0.075)
Observations	1479	1479

**Notes:** ***, **, and *, respectively, indicate that the coefficients are significant at the levels of 1%, 5%, and 10%. Robust standard errors are in parentheses.

While mechanization theoretically diminishes reliance on physical labor and human capital [55], it crucially depends on older adults’ adoption of agricultural social services to offset physical limitations. However, recent evidence has revealed that a surge in service prices, combined with the inherent thriftiness of older adults, has engendered cost resistance [56]. Consequently, this has curtailed older adults’ service use, thereby impeding the effective mitigation of the adverse effects caused by the impact of rural population aging on desirable outputs.

#### 4.3.2. The intermediary effect of agricultural socialized services.

Column (1) of Table 6 shows that the coefficient of rural population aging is positive but statistically insignificant (coefficient = 0.030, p > 0.1), indicating that an increase in the proportion of elderly farmers in households does not significantly promote the adoption of agricultural socialized services. This result contradicts the theoretical expectation that “aging-induced labor shortages would drive demand for labor-substituting services”, and Hypothesis 3 is not supported. The core reason lies in the cost-benefit mismatch of service adoption for elderly farmers. There may be three reasons: First, the high prices of these services strongly deter adoption among older adults [33,56]. Data from the 2019 National Agricultural Product Cost-Benefit Compilation reveal stark contrasts: from 2016 to 2018, three major grain crops showed average net profits of −80.28, −12.53, and −85.59 yuan/mu, compared to machinery service costs of 171.84, 172.03, and 174.27 yuan/mu for the same period. Smallholder farmers, who are predominantly older adults, exhibited a significantly lower likelihood of adopting such services. Second, the low adaptability of service supply to the production characteristics of elderly farmers further reduces adoption motivation. Elderly farmers are more inclined to cultivate small-scale, fragmented plots and mainly grow grain crops with low economic returns. However, current agricultural socialized services are dominated by large-scale, standardized services (e.g., unified mechanized sowing and harvesting), which have high minimum service area requirements (usually ≥0.33 hectares) and are not suitable for fragmented plots. In addition, service providers focus more on production efficiency rather than elderly-friendly operations (e.g., lack of on-site guidance for technology use), which increases the cognitive and operational costs of elderly farmers. Third, the weak risk-bearing capacity of elderly farmers exacerbates their hesitation to adopt services. Elderly farmers’ income is mainly composed of agricultural income and low-level pension benefits, with poor income stability and weak ability to bear potential service risks (e.g., service quality not meeting expectations, crop yield losses due to improper coordination with machinery services). Unlike young farmers who are more willing to try new technologies and services, elderly farmers have a risk-averse preference, and the uncertainty of service effects further reduces their adoption willingness.

**Table 6:** Regression results of mediation effect: agricultural socialized services.

Variable	(1)	(2)	(3)
		low-income groups	high-income groups
Rural population aging	0.030	0.012	0.023*
	(0.029)	(0.083)	(0.013)
Control Variables	YES	YES	YES
Individual fixed effects	YES	YES	YES
Time fixed effects	YES	YES	YES
Observations	1479	740	739

**Notes:** ***, **, and *, respectively, indicate that the coefficients are significant at the levels of 1%, 5%, and 10%. Robust standard errors are in parentheses.

To further verify the robustness of the “cost barrier” mechanism, we conducted a heterogeneous analysis based on household economic conditions (dividing the sample into high-income and low-income groups by the median per capita income). The results are shown in Columns (2) and (3) of Table 6. For low-income households (Column 2), the coefficient is 0.012 and statistically insignificant (p > 0.1), showing no significant promotion effect. For high-income households (Column 3), the coefficient of rural population aging on service adoption is 0.023 and significant at the 10% level (p < 0.1). This indicates that when economic constraints are relaxed, aging significantly promotes the adoption of agricultural socialized services. This heterogeneous result confirms that the cost barrier is the core reason why aging does not drive service adoption, and also explains the contradiction between the empirical result and theoretical expectations: the theoretical expectation ignores the premise that “service adoption requires affordable costs and matching supply,” which is not satisfied for most elderly farmers in China’s rural areas.

Building on the finding that cost barriers prevent aging from driving service adoption, we hypothesize that aging households may instead shift toward an alternative adaptive strategy: farmland reallocation. As aging intensifies and their agricultural management capacity diminishes, farmers often reallocate resources through farmland transfer or abandonment, retaining only limited subsistence farmland [33,57]. Reducing their farmland allows farmers to adopt labor substitution services more flexibly. Therefore, households with a high proportion of older adults may curtail the utilization of agricultural social services by downsizing their farmland. We analyzed the effects of aging on farmland transfer-in and transfer-out, as shown in Columns (1) and (2) of Table 7, to test this hypothesis. In Column (1), where the dependent variable is farmland transfer-out, the coefficient of rural population aging is 0.001 and significant at the 10% level. This indicates that a one-percentage-point increase in aging is associated with a 0.1 percentage point increase in the likelihood of transferring farmland out, reflecting a contraction of operational scale. In Column (2), where the dependent variable is farmland transfer-in, the coefficient is −0.158 and significant at the 5% level. This means that aging significantly reduces the propensity to transfer land in, by 15.8 percentage points for a one-percentage-point increase in aging. Transferred farmland in China primarily flows to new agricultural entities (family farms, cooperatives, and enterprises). Empirical evidence indicates that expanding farmland scale by 1% reduces chemical fertilizer and pesticide use by 0.3% and 0.5%, respectively [58]. Consequently, farmland transfer to these entities enhances AGTFP [59], a conclusion validated by Jin and Wang [29].

**Table 7:** The impact of rural population aging on land allocation.

Variable	Land Transfer-out	Land Transfer-in
Rural population aging	0.001*	−0.158**
	(0.001)	(0.079)
Control Variables	YES	YES
Individual fixed effects	YES	YES
Time fixed effects	YES	YES
Constant	0.011	0.167
	(0.202)	(0.129)
Observations	1479	1479

**Notes:** ***, **, and *, respectively, indicate that the coefficients are significant at the levels of 1%, 5%, and 10%. Robust standard errors are in parentheses.

### 4.4. Analysis *of* heterogeneity

#### 4.4.1. Regional heterogeneity analysis.

Provinces with different levels of economic development exhibited notable disparities in human capital and agricultural green production technology R&D resources, suggesting regional variations in the impact of rural population aging on AGTFP. The sample was divided into eastern, central, and western regions for regional heterogeneity analysis. The regression results are presented in Columns (1)–(3) of Table 8. The results show that the regression coefficients for rural population aging on AGTFP were significantly negative in all three regions, although the magnitude varied. The western region showed the strongest inhibitory effect, followed by the central region, while the eastern region had the weakest effect. This highlights significant regional heterogeneity in the impact of rural population aging on AGTFP. A potential economic explanation is that the eastern region benefits from higher levels of economic development and human capital, as well as widespread agricultural socialized services, which mitigate the adverse effects of aging on AGTFP. In contrast, rural population aging in the central and western regions have lower education levels and insufficient skills, compounded by the underdevelopment of the agricultural socialized service system, which hinders improvements in AGTFP.

**Table 8:** Analysis of heterogeneity regression results.

Variable	Eastern Region	Central Region	Western Region	Pure Elderly Family
	(1)	(2)	(3)	(4)
Rural population aging	−0.010***	−0.689*	−0.928*	−0.282**
	(0.123)	(0.336)	(0.519)	(0.121)
Control Variables	YES	YES	YES	YES
Individual fixed effects	YES	YES	YES	YES
Time fixed effects	YES	YES	YES	YES
Constant	0.922*	2.478***	2.051**	0.920***
	(0.483)	(0.646)	(0.819)	(0.307)
Observations	380	302	304	986

**Notes:** ***, **, and *, respectively, indicate that the coefficients are significant at the levels of 1%, 5%, and 10%. Robust standard errors are in parentheses.

#### 4.4.2. Analysis of heterogeneity in the proportion of aging populations within households.

While aging reduces physical labor capacity and human capital, agricultural production in China is family-based, and the presence of younger laborers in farming households can partially mitigate these constraints. Accordingly, we defined two groups: the experimental group (households with all farmers aged ≥60) and the control group (mixed-age households). The regression results, shown in Column (4) of Table 8, reveal that, compared to households with both older adults and younger laborers, those with only older adult laborers showed stronger aging-induced suppression of AGD, implying that older adults’ physical and cognitive vulnerabilities pose major barriers. Additionally, younger farmers demonstrated significant advantages in offsetting older farmers’ limited ability to acquire new skills and reduce cognitive processing difficulties.

### 4.5. Further analysis

Agricultural mechanization services are an important way to improve farm productivity and substitute manpower in many Asian and African countries [60–62]. Among the existing policy tools, agricultural socialized services are considered a key measure for addressing the issue of “who will farm” in the context of rural population aging in China. However, paradoxically, older adults have not actively adopted agricultural socialized services. This raises a critical research question: Does service adoption alleviate the detrimental effects of aging on AGTFP? To answer this, we conducted an interaction effect analysis by introducing a multiplicative term between rural population aging and socialized service adoption in the regression models.

The results in Column (1) of Table 9 show that the interaction term positively affected AGTFP. This suggests that the use of agricultural socialized services helps alleviate the negative impacts of aging on AGD. Columns (2) and (3) reveal that service adoption enabled older adults to increase their economic and environmental benefits, thereby improving AGTFP. Nevertheless, exorbitant service costs, compounded by older adults’ low technological adaptability and diminished learning capacities, drive widespread service avoidance and perpetuate traditional farming practices. Moreover, faced with labor shortages and rising labor costs, older adults tend to replace machinery and manual labor with fertilizers and pesticides, adopting inefficient “high-volume, low-frequency” application methods [58,59]. This practice exacerbates chemical overuse and substantially lowers green production efficiency. Consequently, cost-reduction strategies and targeted interventions to improve service accessibility and adoption rates among older adults are imperative policy measures for addressing rural population aging.

**Table 9:** Regression results of the further analysis.

Variable	AGTFP	Agricultural Output	Chemical Inputs
	(1)	(2)	(3)
Rural population aging	−0.008***	−0.511**	0.034
	(0.003)	(0.215)	(0.056)
Aging×Services	0.868*	1.559*	−0.010***
	(0.509)	(0.906)	(0.002)
Control Variables	YES	YES	YES
Individual fixed effects	YES	YES	YES
Time fixed effects	YES	YES	YES
Constant	1.262***	0.107	0.204**
	(0.338)	(0.294)	(0.089)
Observations	986	1479	1479

Notes: ***, **, and *, respectively, indicate that the coefficients are significant at the levels of 1%, 5%, and 10%. Robust standard errors are in parentheses.

## 5. Discussion

The aging of populations is accelerating worldwide, presenting significant challenges to multiple global sustainable development goals (SDGs). This demographic shift may lead to critical labor shortages and human capital constraints, particularly in labor-intensive economic sectors. Agriculture as a typical labor-intensive industry could be one of the sectors substantially affected by population aging, especially in countries where smallholder farming is prevalent. The Second National Pollution Source Census indicates that one of the environmental pollution sources within China is agricultural pollution sources. The sector faces dual pressures of ensuring food security while transitioning from resource-intensive practices to environmentally sustainable models. The extensive use of agrochemicals in agricultural production has caused severe environmental pollution. It is indispensable to study the impact and mechanisms of rural population aging on AGD, in order to identify integrated measures.

Using the 2018, 2020, and 2022 CFPS datasets, this study systematically analyzes the impact of rural population aging on AGD. We adopt a micro-level perspective from farmers to construct a comprehensive theoretical framework and explores the specific paths and mechanisms, providing empirical evidence for formulating more differentiated policies. The results of this study serve as a vital reference for nations with a significant number of smallholders.

The study found that rural population aging significantly hinders AGD, primarily by constraining the improvement of economic benefits (agricultural output). Under the smallholder farming structure characterized by geographically dispersed and small-scale plots, labor remains a crucial input factor. The aging of the rural population leads to a decline in both the quantity and quality of agricultural labor, which directly affects the labor input and intensive cultivation practices in grain production, thereby compromising agricultural productivity. The empirical results of this study are consistent with the conclusions of Song et al. [33]. However, compared with Song et al. [33] which adopted provincial-level macro panel data of 31 Chinese provinces from 2000 to 2022 to explore the macro impact mechanism of rural population aging on AGTFP, this study uses micro-level panel data on farmers from the CFPS in 2018, 2020, and 2022, and analyzes the impact of rural population aging on AGD from the perspective of farmers’ production and operational decisions. This makes the research conclusions more practically relevant and reveals the micro-behavioral mechanism underlying the macro-level impact of aging on AGD. Meanwhile, this study decomposes AGD into two dimensions—economic benefits and environmental benefits. We find that the negative impact of rural population aging on AGD stems primarily from the suppression of economic benefits, while its impact on environmental benefits is statistically insignificant. This finding further clarifies the internal structural characteristics of aging’s impact on AGD, supplementing the research dimension not covered by Song et al. [33].

As an important labor-substituting factor, agricultural socialized services have emerged as one of the key pathways to sustain agricultural sustainability and food security in the context of rising rural population aging. This study found that older adults can improve economic and environmental benefits of agricultural production by adopting agricultural socialized services.

Although previous studies suggest that older adults are more motivated to adopt agricultural socialized services, the fundamental assumption is that the benefits of adopting agricultural socialized services outweigh the costs, and that agricultural production is profitable. In practice, however, surging service prices discourage their adoption. Reducing the scale of farmland is a rational choice for maximizing the efficiency of labor force allocation in households during the aging population stage. Farmers often choose to transfer or abandon part of their farmland. Transferred farmland primarily flows to new agricultural entities (family farms, cooperatives, and enterprises). New agricultural entities, tend to have larger farm sizes and are operated by younger farmers, who have a higher average education level. Empirical evidence indicated that expanding farmland scale by 1% reduces chemical fertilizer and pesticide use by 0.3% and 0.5%, respectively [58].

However, it is essential to note that this does not imply that the effects of population aging on agriculture could be effectively addressed through the land transfer. The old-age security system for older adults in rural areas in China still has deficiencies. Although the government has made considerable efforts in recent years to improve rural pension policies, the pension benefits generally remain low and insufficient to fully meet their retirement needs. Consequently, farming income continues to serve as a crucial source of old-age support for many rural households. Many older adults continue farming as long as their health allows, to sustain their basic living. Older adults account for 41.04% of all agricultural laborers and remain an important force in agricultural production in China. Consequently, cost-reduction strategies and targeted interventions to improve service accessibility and adoption rates among older adults are imperative policy measures for addressing rural population aging.

What is more, land transfer in rural China is also subject to notable practical constraints in reality. Remote and fragmented farmland with low yields is often difficult to transfer out. Data from the 2017 and 2019 China Family Panel Studies reveal that 12.1% of rural households abandoned their farmland. This phenomenon not only threatens food security but also contributes to various environmental problems. In addition, farm households continue traditional resource-intensive farming on the remaining land, which is detrimental to the green development of agriculture. Therefore, it is necessary to take corresponding measures from two aspects: the design of the constraint system for farmland abandonment and the cultivation of agricultural socialized service organizations.

## 6. Conclusions and implications

In this study, we use data from the China Family Panel Studies conducted in 2018, 2020, and 2022 to analyze the impact and mechanisms of rural population aging on AGD from a micro-level perspective of farmers. The results indicate that rural population aging significantly hinders AGD, primarily by restricting improvements in economic benefits (agricultural output). In contrast, its effect on environmental benefits (agricultural chemical inputs) is not statistically significant. The adoption of agricultural socialized services by older adults increases agricultural output while reducing chemical inputs, thereby mitigating the adverse effects of rural population aging on AGD. However, surging service prices discourage utilization, prompting farmers to transfer or abandon part of their farmland and continue traditional resource-intensive practices. Furthermore, our analysis of heterogeneous effects reveals significant regional differences, with the impact being more pronounced in the western region than in the central and eastern regions.

Rural population aging leads to severe labor shortages and human capital constraints, resulting in insufficient labor input in agricultural production. In addition, older farmers’ weaker abilities to learn and apply green production technologies may hinder AGD. In theory, employing labor and adopting agricultural mechanization services and other socialized service methods can compensate for deficiencies in labor input and the application of new technologies. However, due to surging service prices, older adults facing severe labor shortages may opt for farmland transfer. Consequently, the smaller scale of self-operated farmland enables them to replace services with personal labor and continue traditional resource-intensive farming. Given that older adults are the mainstay of agricultural production in China and that this trend is likely to intensify in the near future, the agricultural production issues of older adults remain a pressing concern.

The following measures are proposed to promote AGD: First, it is essential to cultivate specialized agricultural socialized service organizations. One feasible approach is for the government to participate in the operation of agricultural economic organizations through equity participation, sponsorship, or guidance. However, it is crucial to clarify the boundaries of governmental power in the operation of these organizations. This ensures stable provision of agricultural socialized services while allowing the market to play a leading role in service supply. In addition, considering factors such as regional economic development levels and farmers’ incomes, tiered or differentiated subsidies should be implemented to provide targeted support for older adults. Second, governments should strengthen land-use regulation and establish mechanisms to curb farmland abandonment. Upgrading infrastructure related to agricultural production can reduce farming costs and reinvigorate farmers’ enthusiasm for cultivation. Finally, human capital development through multichannel approaches should be prioritized to facilitate the adoption of eco-friendly farming technologies. This can be achieved by implementing nationwide green agricultural initiatives, establishing age-appropriate educational systems, and conducting structured training programs. Digital platforms and senior-oriented educational institutions should be used to enhance ecological awareness and technical competencies for sustainable cultivation.

This study had several limitations that should be addressed by future research. First, the regional heterogeneity analysis categorized the provinces into broad geographical divisions (eastern, central, and western China), neglecting intra-regional disparities in policy implementation or resource availability. Second, our evidence was derived solely from China. Future research should conduct cross-national comparative analyses of different levels of rural population aging.

## Supporting information

S1 Data S Data. (XLSX)
